# Erythematous patches and nodules in a neutropenic patient

**DOI:** 10.1016/j.jdcr.2024.06.004

**Published:** 2024-06-10

**Authors:** Angela Lo, Milbrey A. Parke, Caitlin G. Purvis, Kiran Motaparthi

**Affiliations:** a University of Central Florida College of Medicine, Orlando, Florida; b Department of Dermatology, University of Florida, Gainesville, Florida

**Keywords:** chemotherapy, dermatopathology, drug reaction, neutrophilic disorders

A 54-year-old man with progressed high-grade myelodysplastic syndrome admitted for chemotherapy developed several painful skin lesions on day 15 of treatment with daunorubicin and cytarabine. Exam demonstrated several erythematous plaques with induration, warmth, and tenderness on the legs and a tender, erythematous nodule on the forearm (Fig 1). Labs were notable for severe pancytopenia, including a leukocyte count of 2.6 × 10^3^ cells/uL and absolute neutrophil count of 730 cells/mL. Biopsies were obtained for histopathology (Figs 2 and 3). Tissue culture was sterile.

**Fig 1:**
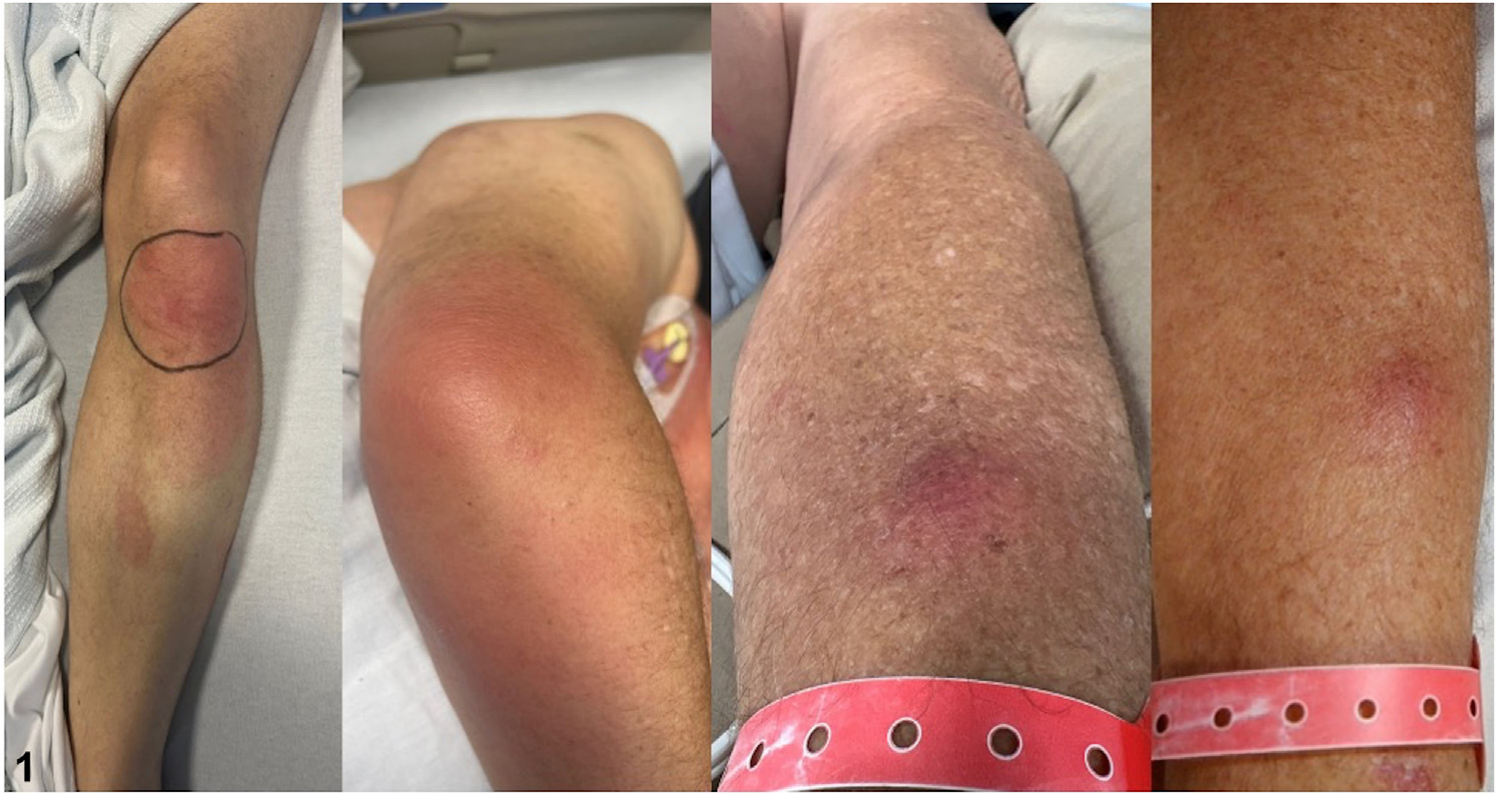

**Fig 2:**
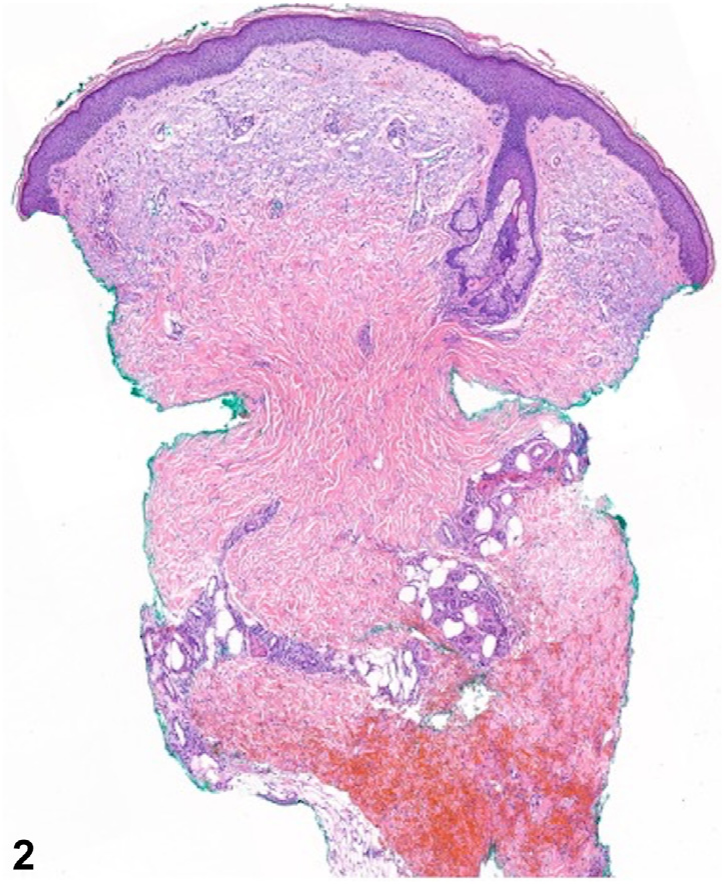

**Fig 3:**
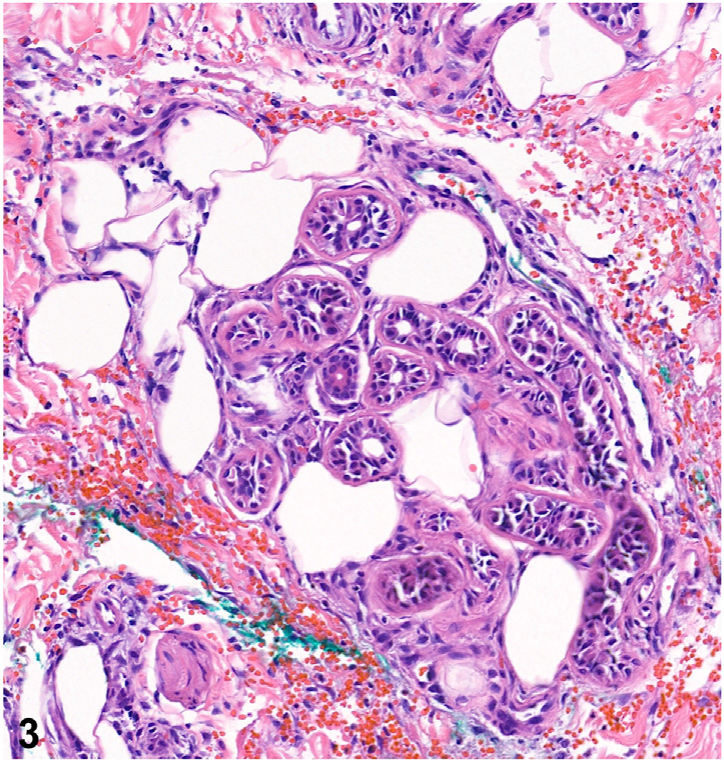

**Question 1: Which of the following is the most likely diagnosis?**

A. Neutrophilic eccrine hidradenitis (NEH)
B. Leukemia cutis
C. Acute febrile neutrophilic dermatosis (Sweet Syndrome)
D. Subcutaneous mycosis
E. Erythema nodosum

**Answers:**

A. NEH – Correct. NEH is a rare and self-limiting neutrophilic dermatosis that presents with tender, erythematous, and edematous plaques and nodules developing 1-2 weeks following chemotherapy, most commonly cytarabine. Histopathology reveals a perieccrine inflammatory infiltrate with neutrophils and lymphocytes, as well as edema, necrosis, and squamous metaplasia of eccrine coils. In neutropenic patients, inflammation may be sparse, but characteristic eccrine changes are maintained.^1^
B. Leukemia cutis – Incorrect. Leukemia cutis often presents as asymptomatic nodules and papules favoring the trunk, extremities, and head. Histopathology shows infiltration of neoplastic leukocytes or their precursors into the dermis and subcutis.
C. Acute febrile neutrophilic dermatosis (Sweet Syndrome) – Incorrect. Sweet syndrome is the most common neutrophilic dermatosis and is associated with malignancies, including myelodysplastic syndrome. However, an abrupt onset of multiple, tender, erythematous plaques, or nodules is characteristic, and histopathology would reveal marked edema and a significant dermal neutrophilic infiltrate without eccrine gland pathology.
D. Subcutaneous mycosis – Incorrect. The development of a solitary, indurated, subcutaneous nodule following transcutaneous trauma warrants concern for a deep fungal infection, and eccrine gland necrosis can be seen. However, fungal organisms would be identified on histopathology and tissue culture.
E. Erythema nodosum – Incorrect. While clinical findings may imitate NEH, erythema nodosum mainly localizes on the extensor surface of the legs. Histopathology would reveal a septal granulomatous panniculitis without vasculitis.

**Question 2: Which of the following conditions is most commonly associated with neutrophilic eccrine hidradenitis?**

A. Solid tumors
B. Bacterial infection
C. Hematologic malignancy
D. HIV
E. Systemic lupus erythematosus (SLE)

**Answers:**

A. Solid-tumors – Incorrect. NEH can be considered a reactive disorder and is commonly associated with malignancies. However, solid tumors such as testicular carcinoma, lung carcinoma, breast carcinoma, and osteosarcoma have been only rarely described in association.
B. Bacterial infection – Incorrect. While bacterial pathogens including *Serratia*, *Staphylococcus*, *Enterobacter*, and *Nocardia* have been reported in patients with clinicopathologic features of NEH, this is not the most common cause. When considering the diagnosis of NEH, infection should be excluded with sterile tissue cultures.
C. Hematologic malignancy – Correct. NEH was originally described in acute myelogenous leukemia^1^ and has also been reported in chronic lymphocytic leukemia, Hodgkin disease, and non-Hodgkin lymphoma. NEH is proposed to be a consequence of direct cytotoxicity by drugs secreted in eccrine epithelia.
D. HIV – Incorrect. Although there have been case reports of HIV-associated NEH,^2^ 90% of cases develop in patients with underlying malignancies. The sporadic occurrence of NEH in HIV-infected patients suggests an eccrine pathology similar to that described with chemotherapy but mediated by either HIV or antiretroviral treatments.
E. SLE – Incorrect. Neutrophilic dermatoses are an uncommon manifestation of lupus. Most neutrophilic infiltrates in the setting of SLE are associated with bullous SLE or vasculitis.

**Question 3: Which of the following treatments is initially preferred?**

A. Nonsteroidal anti-inflammatory drugs
B. Corticosteroids
C. Dapsone
D. Colchicine
E. Adalimumab

**Answers:**

A. Nonsteroidal anti-inflammatory drugs – Correct. NEH is generally a self-limiting condition that does not require therapy, with most cases resolving within a month of chemotherapy cessation. However, supportive care, including symptomatic management of fever and or pain with NSAIDs, is recommended.
B. Corticosteroids – Incorrect. Corticosteroids, both systemic and topical, have been shown to shorten the duration of lesions and relieve pain in patients with refractory disease. However, the need for systemic steroids in NEH is debatable, and high-dose systemic steroids should be used with caution in neutropenic patients.
C. Dapsone – Incorrect. Case reports have shown that dapsone, which decreases neutrophil migration, can be beneficial in treating NEH, but this is generally reserved for recurrent disease.^3^
D. Colchicine – Incorrect. Colchicine decreases neutrophil chemotaxis, adhesion, and degranulation and has successfully treated neutrophilic dermatoses including Behcet disease and Sweet syndrome. Colchicine has been efficacious in some cases of NEH but is not a first-line therapy.^4^
E. Adalimumab – Incorrect. Adalimumab is a TNF-alpha inhibitor that is often utilized for the treatment of hidradenitis suppurativa, pyoderma gangrenosum, rheumatoid arthritis, and psoriasis. It is not an appropriate therapy for NEH.

## Conflicts of interest

None disclosed.
